# 14820 Mental Health Mobile App Use in Integrated Primary Care Settings: Considerations for Serving Underserved Patients

**DOI:** 10.1017/cts.2021.534

**Published:** 2021-03-30

**Authors:** Shinobu Watanabe-Galloway, Maggie Emerson, Danae Dinkel, Jennifer Caspari-Harsh, Josiane Kabayundo, Louis Fok, David Johnson

**Affiliations:** University of Nebraska Medical Center - UNMC

## Abstract

ABSTRACT IMPACT: Mobile app may help improve the depression symptoms among underserved patients OBJECTIVES/GOALS: Depression is one of most common mental health conditions and the leading cause of disability worldwide, affecting about one in 10 adults in the US. The aim of this study was to explore the factors that affect feasibility of incorporating mobile app self-management tools for depression in integrated primary care settings. METHODS/STUDY POPULATION: This was a cross-sectional questionnaire study of depressed patients at two primary care clinics in a Midwest academic medical center. Adult patients (≥19 years) who had an active or previous diagnosis of depression were included in the study. A self-administered survey collected information pertaining to demographics, smartphone ownership, data plan type, smartphone application usage, mobile app self-management interest, health literacy, and patient activation. Chi-square analysis was conducted to compare the patient demographic characteristics, the smartphone ownership, phone plan, smartphone use for health information between two clinics. Multinominal logistic regression analysis was conducted to examine the association between the patient activation and patient characteristics. RESULTS/ANTICIPATED RESULTS: Over 80% of patients owned a smartphone, 80.5% were willing to use data for depression management, and 68.9% believe an app can help in depression management. A higher literacy level was significantly associated with higher level of patient activation (Chi-square=8.5453; p=0.0360). These results suggest that planning interventions that use mobile apps within this patient population is likely feasible and the intended underserved patients at these clinics have an interest in using depression related apps which is similar to findings found by other studies exploring app interest. DISCUSSION/SIGNIFICANCE OF FINDINGS: Understanding patient activation levels within a given population can help to shape corresponding needs. The use of depression related self-management mobile apps will likely require the development of educational materials to facilitate patient use and engagement which means understanding the literacy needs of this population as well.

